# Two homologous *Salmonella* serogroup C1-specific genes are required for flagellar motility and cell invasion

**DOI:** 10.1186/s12864-021-07759-z

**Published:** 2021-07-05

**Authors:** Xiujuan Zhou, Bin Liu, Yanhong Liu, Chunlei Shi, Pina M. Fratamico, Lida Zhang, Dapeng Wang, Jianhua Zhang, Yan Cui, Ping Xu, Xianming Shi

**Affiliations:** 1 MOST-USDA Joint Research Center for Food Safety, School of Agriculture & Biology, and State Key Lab of Microbial Metabolism, Shanghai Jiao Tong University, Shanghai, 200240 China; 2 College of Food Science and Engineering, Northwest Agriculture and Forestry University, Yangling, 712100 Shaanxi China; 3 Molecular Characterization of Foodborne Pathogens Research Unit, Eastern Regional Research Center, Agricultural Research Service, U.S. Department of Agriculture, 600 East Mermaid Lane, Wyndmoor, PA 19038 USA

**Keywords:** *Salmonella*, Serogroup C1, RNA-Seq, Motility, Virulence, Growth, Choleraesuis

## Abstract

**Background:**

*Salmonella* is a major bacterial pathogen associated with a large number of outbreaks of foodborne diseases. Many highly virulent serovars that cause human illness belong to *Salmonella* serogroup C1, and *Salmonella* ser. Choleraesuis is a prominent cause of invasive infections in Asia. Comparative genomic analysis in our previous study showed that two homologous genes, *SC0368* and *SC0595* in *Salmonella* ser. Choleraesuis were unique to serogroup C1. In this study, two single-deletion mutants (Δ0368 and Δ0595) and one double-deletion mutant (Δ0368Δ0595) were constructed based on the genome. All these mutants and the wild-type strain were subjected to RNA-Seq analysis to reveal functional relationships of the two serogroup C1-specific genes.

**Results:**

Data from RNA-Seq indicated that deletion of *SC0368* resulted in defects in motility through repression of σ^28^ in flagellar regulation Class 3. Consistent with RNA-Seq data, results from transmission electron microcopy (TEM) showed that flagella were not present in △0368 and △0368△0595 mutants resulting in both swimming and swarming defects. Interestingly, the growth rates of two non-motile mutants △0368 and △0368△0595 were significantly greater than the wild-type, which may be associated with up-regulation of genes encoding cytochromes, enhancing bacterial proliferation. Moreover, the △0595 mutant was significantly more invasive in Caco-2 cells as shown by bacterial enumeration assays, and the expression of lipopolysaccharide (LPS) core synthesis-related genes (*rfaB*, *rfaI*, *rfaQ*, *rfaY*, *rfaK*, *rfaZ*) was down-regulated only in the △0368△0595 mutant. In addition, this study also speculated that these two genes might be contributing to serotype conversion for *Salmonella* C1 serogroup based on their apparent roles in biosynthesis of LPS and the flagella.

**Conclusion:**

A combination of biological and transcriptomic (RNA-Seq) analyses has shown that the *SC0368* and *SC0595* genes are involved in biosynthesis of flagella and complete LPS, as well as in bacterial growth and virulence. Such information will aid to revealing the role of these specific genes in bacterial physiology and evolution within the serogroup C1.

**Supplementary Information:**

The online version contains supplementary material available at 10.1186/s12864-021-07759-z.

## Background

*Salmonella* infections in humans and animals are a global public health problem [1]. The genus *Salmonella* is comprised of two species, *Salmonella bongori* and *Salmonella enterica.* Of the six *S. enterica* subspecies, subspecies I accounts for more than 99.5% of isolated *Salmonella* strains. The serogroup classification after the subspecies level relies on differences in the surface O-antigens, and the individual serovar is distinguished by flagellar H-antigens and biochemical tests [2]. There are > 2600 *Salmonella* serotypes, and some of the more virulent serovars are within *Salmonella* serogroup C1 [3]. All serovars in this serogroup share the same O_7_ antigen. For example, *Salmonella* ser. Choleraesuis (O_6,7_:H_c,1,5_) was originally isolated from pig intestines and causes extra-intestinal or focal infections in humans. This serovar is associated with a higher mortality rate compared to other *Salmonella* serovars [4]. *Salmonella* ser. Paratyphi C (O_6,7_[Vi]:H_c,1,5_) is a human-restricted pathogen that causes paratyphoid fever, a serious and potentially fatal systemic infection [5]. *Salmonella* ser. Infantis (O_6,7,14_:H_r,1,5_) typically causes gastroenteritis and is the fourth most prevalent serovar causing human infections in Europe [6].

In our previous study, seven conserved and specific genes in *Salmonella* serogroup C1 were identified by comparative genomic analysis [7]. These C1-specific genes mainly encoded membrane proteins with high numbers of transmembrane segments (TMS). Five of the C1-specific genes are located in the C1 *rfb* gene (O-antigen gene) cluster that includes a putative flippase (*SC2092*) and polymerase (*SC2098*) for the O_7_-antigen [8, 9]. The other two genes (*SC0368* and *SC0595*) that located outside of the O-antigen gene cluster are highly homologous (83%) to each other, which encode hypothetical proteins with 10 TMS [7]. Currently, no related research on these two homologous genes is available. Notably, genes downstream of *SC0368* are *gtrB* (encoding a glycosyl transferase) and *gtrA* (encoding a bactoprenol-linked glucose translocase) [10, 11]. Downstream genes of *SC0595* are *yfdH* and *rfbI*, which are putative orthologs of *gtrA* and *gtrB*, respectively [12, 13]. These *gtrABC* genes are located outside the main O-antigen chromosomal gene clusters and are associated with a bacteriophage involved in serotype conversion via O-antigen glucosylation [10, 14]. The *yfdH* and *rfbI* genes are also found upstream of *SC0594* but are oriented in the opposite direction. These observations suggested that the two homologous C1-specific genes might have evolutionary relationships associated with serotype conversion.

One of the C1-specific genes, *SC2092*, is associated with biosynthesis of the O-antigen of lipopolysaccharide (LPS) [8]. In addition, an *SC2092* null mutant displayed a NaCl-dependent deficiency in motility and produced fewer flagella than the wild-type strain [8]. Since *SC0368* and *SC0595* are also C1-specific genes, we wished to determine if the two orthologous genes *SC0368* and *SC0595* affect LPS biosynthesis and motility in *Salmonella*. To test this hypothesis, in-frame single (△0368 and △0595*)* and double (△0595△0368*)* deletion mutants were constructed using *Salmonella* ser. Choleraesuis ATCC 10708 as the parental strain. We used a combination of biological and transcriptomic (RNA-Seq) analyses to determine whether these genes are necessary for bacterial growth, motility, cell invasion and LPS biosynthesis. Information from this study will aid in evaluation of the effects of these genes on bacterial structure and physiology, and will assist in identifying their role in serotype conversion in serogroup C1.

## Results

### Differentially expressed genes (DEGs) in three mutants

To reveal functional relationships of the two orthologous *Salmonella* serogroup C1-specific genes *SC0368* and *SC0595*, we constructed single (Δ0368 and Δ0595) and double (Δ0368Δ0595) deletion mutants (Figure S1) and used these strains for RNA-Seq analysis. The sequencing yielded more than 28 million reads for 12 samples and 98.83–99.58% of the high-quality reads were mapped to the reference genome sequence consisting of 4500 *Salmonella* genes (Table S1). According to analysis with the DEGseq software, 75, 80, and 211 genes were considered differentially expressed in the △0368, △0595, and △0368△0595 mutants compared to the wild-type strain, respectively (> 2-fold changes, FDR < 0.05). There were 12 genes in strain △0368 and 35 in strain △0368△0595 that showed > 10-fold down-regulation. On the other hand, 2 genes in strain △0595 and 1 in strain △0368△0595 were > 5-fold up-regulated (Table 1).

**Table 1:** Statistics of differentially expressed genes in three mutants compared to wild type

Mutants	Down-regulated genes (number)		Up-regulated genes (number)	
	> 2-fold	> 10-fold	> 2-fold	> 5-fold
△0595	38	0	42	2
△0368	37	12	38	0
△0368△0595	139	35	72	1

**Fig. 1:**
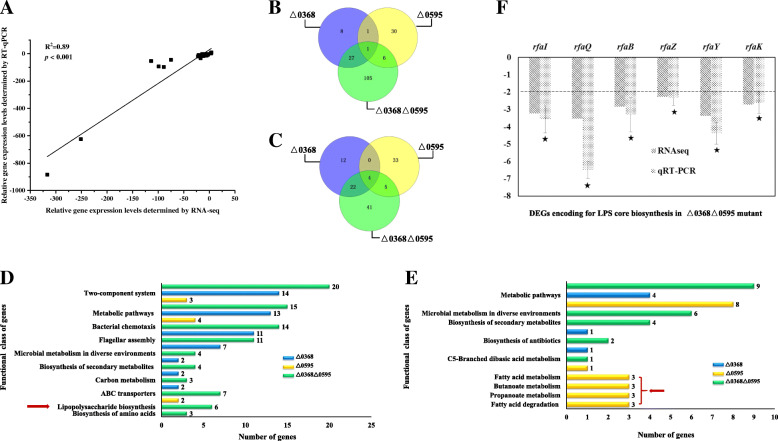
Differential gene expression of *SC0368* and *SC0595 Salmonella* ser. Choleraesuis mutants. **A** Correlation of RT-qPCR and RNA-Seq results for 18 differentially expressed genes between the △0368△0595, △0595, △0368 mutants and the parental wild-type strain ATCC 10708 (*R*^2^ = 0.89, *p* < 0.001). Distributions of down-regulated genes (**B**) and up-regulated genes (**C**) with at least a 2-fold change in the mutants relative to the wild-type strain. **D** Down-regulated KEGG pathways. **E** Up-regulated KEGG pathways. Blue, mutant △0368; Yellow, mutant △0595 and Green, mutant △0368△0595. **F** Repression of DEGs encoding for LPS core synthesis (marked with red arrow in **D**) in strain △0368△0595

To validate the RNA-Seq results, a total of 18 up- and down-regulated genes derived from the three different mutants were selected for RT-qPCR analysis (Fig. 1 and Table S2). As shown in Fig. 1A, RT-qPCR data correlated well with RNA-Seq results (*R*^2^ = 0.89) with a *p*-value < 0.001, indicating that the RNA-Seq data were very reliable.

A Venn diagram was used to visualize the distribution of DEGs in each deletion mutant. The *fli*A gene encoding a flagellar biosynthesis sigma factor (σ^28^) was down-regulated in all three mutants (Fig. 1B and Table S3). There were 4 genes that were up-regulated in all three mutants, including *SCTRNA84* and *SCTRNA85* (both encoding tRNAs), *SC3193* (encoding a hypothetical protein), and *glgS* (encoding a glycogen synthesis protein) (Fig. 1C and Table S4).

Down-regulated genes in the △0368△0595 mutant (Table S3) were classified into 10 functional KEGG categories (Fig. 1D, *p* < 0.05). The only genes shared between strains △0368 and △0368△0595 fell within 5 functional classes, including chemotaxis, flagellar assembly, microbial metabolism, biosynthesis of secondary metabolites, and carbon metabolism (Fig. 1D, Table S3). There were 4 enriched pathways (propanoate metabolism, butanoate metabolism, fatty acid degradation, and fatty acid metabolism) in up-regulated genes only found in the △0595 mutant (Fig. 1E, red arrow). In addition, 6 genes involved in synthesis of the LPS core component were down-regulated only in the double deletion mutant Δ0368Δ0595 (Fig. 1D red arrow, and Table S3) and RT-qPCR assays confirmed that genes *rfaB*, *rfaI*, *rfaQ*, *rfaY*, *rfaK* and *rfaZ* were > 2-fold down-regulated (from 2.6 to 6.5) only in the △0368△0595 mutant (Fig. 1F, Table S2).

### Deletion of *SC0368* resulted in defects in flagellar motility

**Fig. 2:**
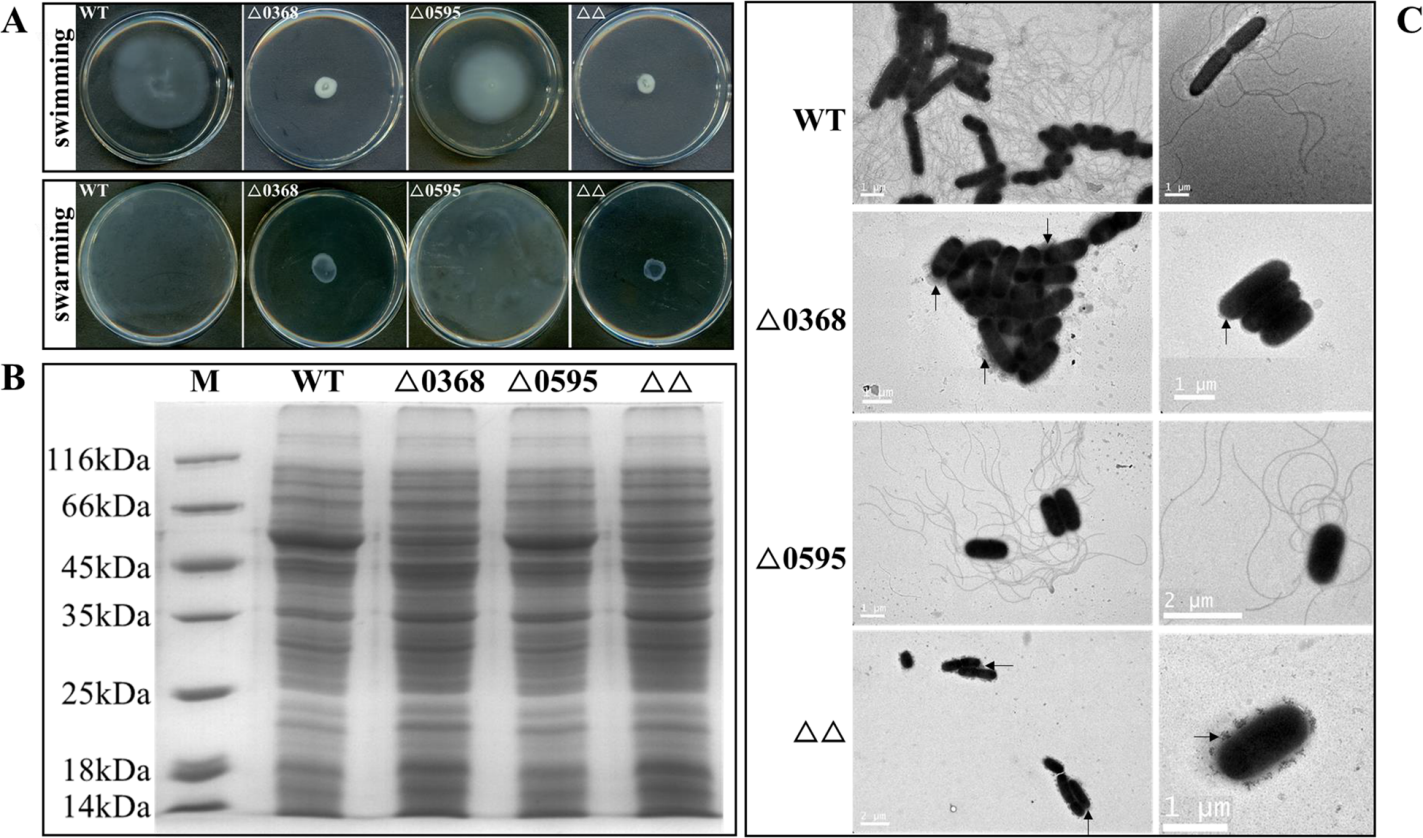
Bacterial motility and flagellar biosynthesis of deletion mutants compared to wild-type (WT). **A** The photos of one out of six independent experiments was randomly selected to show the motility behaviors of the indicated strains (**B**) SDS-PAGE analysis of bacterial total proteins (**C**) TEM photomicrographs of flagella and bacterial morphological features. Some spherical structures seem like outer membrane blebs were marked with black rows in these two flagella defect mutants

The wild-type strain and △0595 mutant both showed a high degree of motility by both swimming and swarming assays (Fig. 2A). However, this was not the case for △0368△0595 and △0368 mutants, which had not spread far from the inoculation point (Fig. 2A), indicating that an in-frame deletion of the *SC0368* gene significantly reduced motility relative to the wild-type strain. Total proteins of the wild-type and the three mutant strains were separated by sodium dodecylsulphate polyacrylamide gel electrophoresis (SDS-PAGE) (Fig. 2B). A band with size at ~ 50–52 kDa, likely representing a flagellin protein [15], was absent in the △0368△0595 and △0368 mutants (Fig. 2B). Furthermore, TEM analysis indicated that the non-motile mutants △0368△0595 and △0368 lacked flagella compared with the wild-type and △0595 strains (Fig. 2C). Moreover, these two flagella defect mutants appeared to have outer membrane blebs that were not seen in wild-type or △0595 mutant (Fig. 2C, marked with black rows), indicating that the outer membrane is less stable in the absence of *SC0368*.

### Bacterial growth rates were increased in *SC0368* deletion mutants

**Fig. 3:**
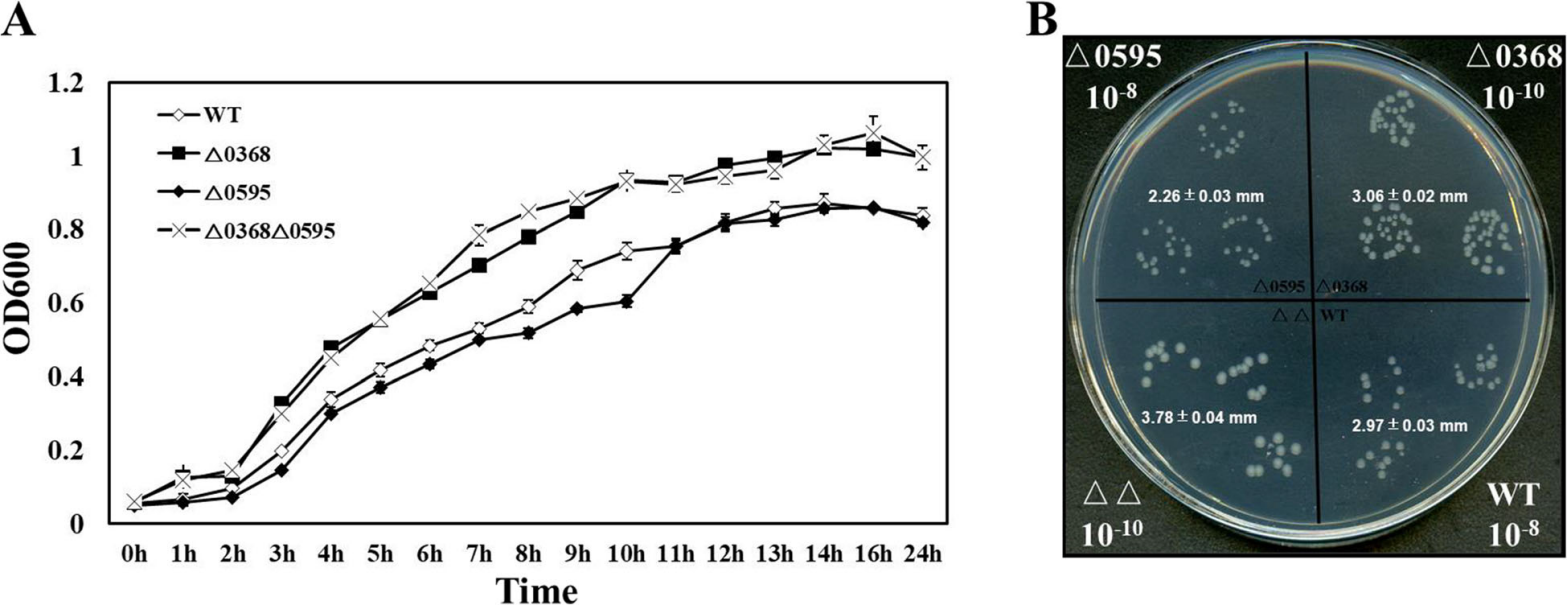
The growth of the wild-type strain and mutants in culture. **A** Growth curves, error bars indicate that each experiment was repeated six times and **B** colony cultures of three deletion mutants and the wild-type strain (WT) at 12 h. The average size of the bacterial colonies for each strains was marked in panel B

The growth rates of the wild-type strain and three mutants were investigated to determine whether the differences in growth were caused by deletion of the *SC0368* and *SC0595* genes*.* Interestingly, the two non-motile mutants △0368 and △0368△0595 grew significantly faster and to a higher yield than the motile strains by cell counts (*p* < 0.05, *n* = 6) (Fig. 3A). The average size of bacterial colonies for △0368 or △0368△0595 was larger than that of △0595 strain at 12 h (Fig. 3B). Specifically, △0368△0595 strains formed the largest colonies at 3.78 ± 0.04 mm, while the smallest were for △0595 strains at 2.26 ± 0.03 mm.

### *SC0595* and *SC0368* are related with invasion capacity in Caco-2 cells

**Fig. 4:**
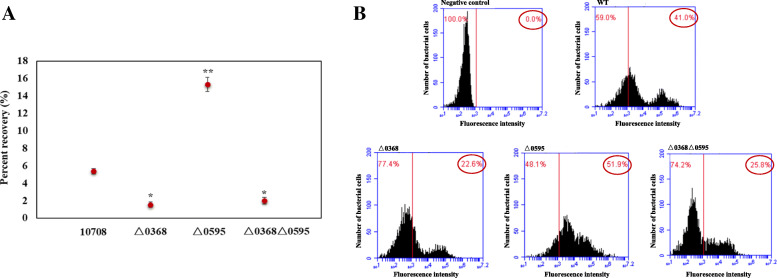
Caco_2_ cell invasion analysis by plate counting and flow cytometry. **A** Experiments were performed in duplicate and repeated three times. Statistical analyses of the invasive capacity compared to wild-type (WT) were performed using the SPSS 13.0 for Windows software; a *p* < 0.05 was considered to be significant, marked with * and *p* < 0.01 was considered to be extremely significant, marked with **; **B** Negative control is the same volume of bacterial suspension without simultaneous staining treatment by SYTO 9, and the value of 10^3^ was chosen as the cut-off for cell invasion because 100% unstained bacteria were covered. Red cycle: the percentage of live invasion bacteria cells labelled by SYTO 9 fluorescence at above 10^3^

Both plate counting and flow cytometry were used to test the invasion capacity in Caco-2 cells of the three mutants and the wild-type strain. The results from plate counting indicated that the △0595 mutant exhibited the greatest invasion capacity with ~ 15.0% recovery rate, and the invasion capacity of the △0595 mutant was significantly higher than that of the wild-type strain (*p* < 0.01, Fig. 4A). On the other hand, a significantly lower invasion capacity with < 2.5% recovery rate was observed of the *SC0368* deletion mutants (~ 1.8% for △0368 and ~ 2.4% for △0368△0595, respectively) compared with the wild-type strain (*p* < 0.05, Fig. 4A). Similar results were confirmed by bacterial cell enumeration using flow cytometry (Fig. 4B). The fluorescence value of 10^3^ was chosen as the cut-off for dye-bacteria binding because 100% unstained bacteria were covered at this point in the negative control group. In 100 μL of cell lysates, the percentage of bacterial cells strained by SYTO 9 was 41.0, 22.6, 51.9, and 25.8% for wild-type, △0368, △0595, and △0368△0595, respectively, which was considered as the cell invasive capacity of each stain. In other words, the cell invasion capacity increased with the deletion of *SC0595* (△0595), weakened with deletion of *SC0368* (△0368 and △0368△0595).

### Recovery of motility, growth, and cell invasion with complementation of the three mutants

**Fig. 5:**
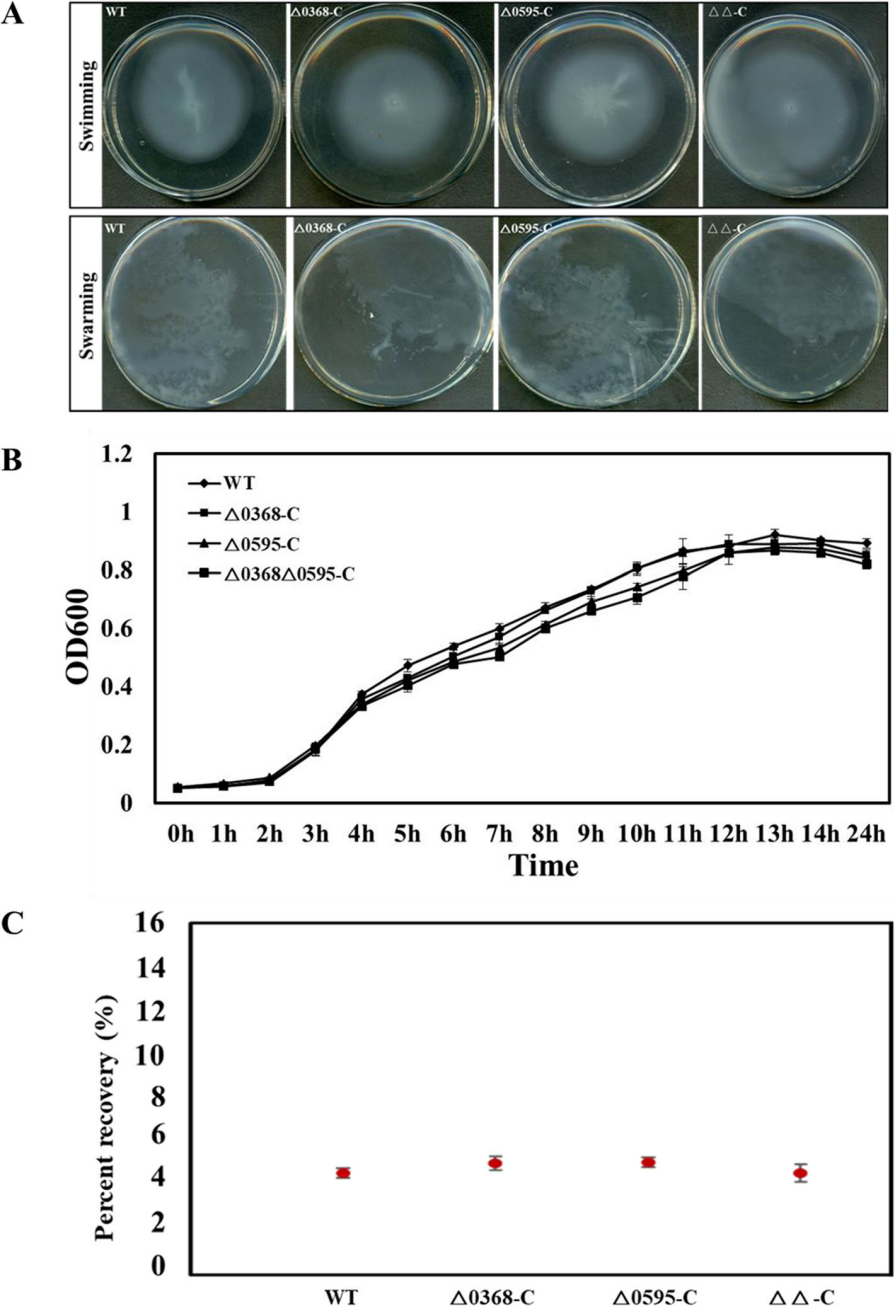
Data on flagellar motility, bacterial proliferation and cell invasion of complementary strains relative to wild-type strain

To demonstrate if the *SC0368* or *SC0595* mutation alone was responsible for the observed phenotypes, three complemented strains (△0368-C, △0595-C, and △0368△0595-C) were generated. Compared with the wild-type strain, the motility defect and increased growth did not occur in the △0368-C and △0368△0595-C strains (Fig. 5A and B). Meanwhile, the invasion capacity of these three complemented strains (△0368-C, △0595-C, and △0368△0595-C) were at a similar lever with the wild-type strain at 4.6 ± 0.4% ~ 5.1 ± 0.2% recovery rate (Fig. 5C).

## Discussion

During the past 10 years, comparative genomics has been used by our team to mine conserved and specific markers for *Salmonella enterica* [16] at the species [17, 18], serogroup [7] and serotype [19] levels. Based on these markers, rapid identification methods including multiplex PCR [7, 19], real-time PCR [17], and real-time reverse-transcriptase PCR [20] methods have been consecutively developed. Since all these specific markers have been verified with a large number of *Salmonella* strains, these PCR detection systems showed high specificity, which has been recognized by experts in other research teams [21, 22].

Recently, we investigated the specific biological functions and roles in evolution of these verified detection markers by using bioinformatics and molecular biological methods [8, 18]. GO (Gene Ontology) clusters analysis has shown that species-specific detection markers are significantly enriched in pathogenesis and the type III secretion system [18]. Most of the specific markers for *Salmonella* ser. Enteritidis, an important serotype frequently found in contaminated eggs, are predicted phage-related proteins [19], some of which have been proven to be necessary in bacterial survival in egg white [23]. The majority of serogroup-specific markers are located in the *rfb* gene cluster that is associated with LPS biosynthesis by sugar metabolism or glycosyl and O-actetyl transfer [7]. Furthermore, we have discovered that the C1-specific gene, *SC2092*, was involved in flagellar motility [8]. In the current study, the two homologous C1-specific genes (*SC0368* and *SC0595*) that are located outside the *rfb* gene cluster, were observed to have important roles in flagella motility, bacterial growth, and cell invasion.

### The motility defect was due to the reduced expression of flagellar class 3 genes regulated by σ^28^

**Fig. 6:**
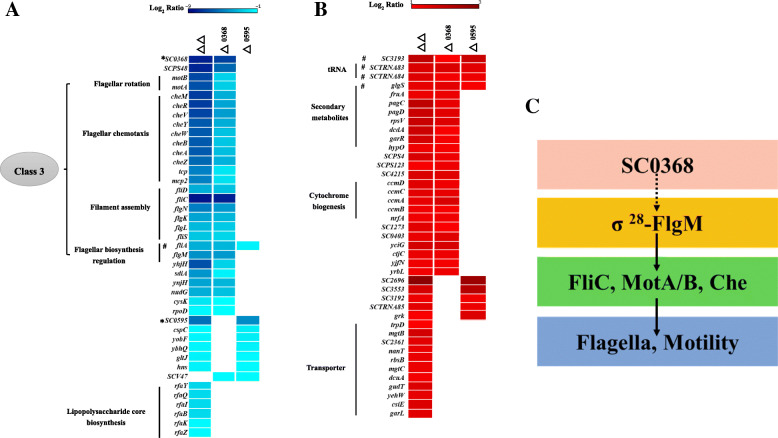
Heat map of differentially expressed genes and schematic diagram of SC0368 in bacterial motility regulation. **A** Down-regulated genes. **B** Up-regulated genes. Each column represents the different mutants as indicated. Each row represents individual gene. Scale is log2fold changes ranging from − 9 (dark blue) to − 1 (cyan) and from + 1 (red) to + 3 (dark red) relative to wild-type, respectively. * Deleted gene. ^#^ DEGs shared in three mutants. **C** The regulation of SC0368 in bacterial motility

Previous researches have shown that more than 60 genes are involved in flagellar regulation in *Salmonella*, and they are divided into three classes: class 1 (early), class 2 (middle), and class 3 (late) according to their temporal expression after induction of the flagellar regulon [24, 25]. The class 3 promoters transcribe the late genes for filament assembly, flagella rotation, and chemotaxis [26]. Genes whose expression is required late in flagellar assembly are primarily transcribed by FliA [24]. In the current study, a total of 20 motility-associated genes that fell within 2 functional classes (chemotaxis and flagellar assembly) were significantly repressed in the non-motile strains (Fig. 1D, Table S3). Significantly, this group of 20 down-regulated genes in the current study (Fig. 6A, Tables S2 and S3) that included *fliA* encoding sigma factor σ^28^ and *flgM* encoding its cognate anti-σ-factor are required for class 3 transcription [26, 27]. Therefore, both the transcriptomic (Fig. 6A, Tables S2 and S3) and biological data (Fig. 2) demonstrated that deletion of *SC0368* resulted in defects in motility through repression of σ^28^ in flagellar regulation Class 3 (Fig. 6C). On the other hand, *fliA* gene had a less amount of reduction (~ 3 fold in both RNAseq and RT-qPCR assays) in △0595 mutant and no other flagella related genes had differential expression that there was no difference with wild-type in flagella and motility. It is hard to explain from the current data how these two membrane proteins with 10 TMS encoded by *SC0368* and *SC0595* could affect the expression of σ^28^. There is a possibility that these signaling linkages might be through a regulatory system sensing envelope stress just like Cpx and σ^E^ [28], and the differences of the structure and the expression level between SC0368 and SC0595 would diversify stress-sensing response. Notably, some outer membrane blebs might appear in the *SC0368* deletion mutants (Fig. 2C), suggesting that the homeostasis of the bacterial cell membrane has been damaged or reformed by some unknow stress-sensing mechanisms.

### The RNA-Seq data give clues to explain the increase in growth with deletion of *SC0368*

The RNA-Seq data showed that all 4 genes in the heme ABC exporter operon (*ccmA*, *ccmB*, *ccmC,* and *ccmD*), encoding cytochrome C [29] and 1 gene (*nrfA*) encoding cytochrome c552 nitrite reductase [30] were up-regulated in both the △0368 and △0368△0595 mutants (Fig. 6B and Table S4). These genes participate in a variety of biological processes, including oxygen transport, oxygen binding, electron transfer, and transcriptional regulation [31, 32]. Enhanced proliferation resulting from the up-regulation of these genes has been reported for *Campylobacter jejuni* [33] and *Salmonella* ser. Typhimurium [34]. Furthermore, the *rpsV* gene that showed increased expression in both the △0368 and △0368△0595 mutants (Fig. 6B and Table S4) is a 30S ribosomal subunit that is a growth regulator during stationary phase [35]. Likewise, there is another possibility that the growth advantage observed in nonmotile mutants may be simply due to the lack of energy consumption to make flagella, because flagella biosynthesis is an energy intensive process. Additionally, a previous report has shown that the mutant of FliA in *E. coil* elevated fitness by increasing growth rates and reducing flagellar transcription, which is similar with these flagella defect mutants (△0368 and △0368△0595) in this current study that the gene expression of *fliA* was significantly inhibited.

### The double deletion of *SC0368* and *SC0595* genes may affect LPS core biosynthesis

Results from protein tertiary structure predictions (http://topcons.cbr.su.se/) based on the deduced amino acid sequence of SC0368 or SC0595 indicated that these putative proteins shared similarity with LPS biosynthesis related proteins. SC0368 shares 11.90% identity to the LPS core biosynthesis protein RfaG, and SC0595 shares 17.16% identity to the oligosaccharide transferase that is essential for side branch synthesis of the core oligosaccharide [36]. Furthermore, the known or putative functions of these 6 down-regulated genes (i.e. *rfaB*, *rfaI*, *rfaQ*, *rfaY*, *rfaK* and *rfaZ*) only in the double deletion mutant Δ0368Δ0595 are involved in LPS core biosynthesis (Fig. 1D red arrow, Fig. 6A and Table S3, marked with a). This suggests that *SC0368* and *SC0595* probably have synergistic effects on LPS core synthesis. In other words, the existence of these two highly homologous genes in the *Salmonella* ser*.* Choleraesuis genome may ensure the complete synthesis of LPS.

LPS in most Gram-negative bacteria consists of the lipid A moiety linked to the short core oligosaccharide and the distal O-antigen polysaccharide chain [37]. Several research groups have described diverse effects on motility in those LPS biosynthesis defect mutant strains such as in *Proteus mirabilis* [38], *Salmonella* [39] and *E. coli* [40]. Our previous results have shown that mutation of a *Salmonella* serogroup-C1-specific gene (*SC2092*) abrogated O-antigen biosynthesis and displayed motility deficiency due to lower flagella synthesis [8]. Results of the present study also showed that the two serogroup-C1-specific genes probably affected LPS core oligosaccharide synthesis (Fig. 1D red arrow, Fig. 1E and Table S3, marked with a) and flagellar motility. Therefore, these serogroup-specific genes may participate in maintaining bacterial cell integrity by controlling the synthesis of complete LPS and flagella.

Notably, these 6 down-regulated genes encoded proteins associated with synthesis of the side branch of the core oligosaccharide [9]. These included RfaY that adds phosphate groups to the second Hep residues and RfaB that adds a Gal residue to the first Glc of the second Hep residue, as well as RfaI that adds the third Glc residues to the second Glc [12, 41]. However, agglutination tests with O_7_ antibody in the △0368△0595 mutant were positive and no apparent changes in the LPS profiles was found in the wild-type and three mutants (data not shown); probably because the proteins encoded by the 6 down-regulated genes were not the binding sites of the O-antigen [42], or this repressed effect on the LPS core was not sufficient for detectable phenotypic changes. Therefore, more detailed studies are needed to prove if the quantity and integrity of synthesized LPS are affected in these deletion mutants.

### The evolution significances of two homologous genes (*SC0368* and *SC0595*) with different phenotypes

Previous studies have provided evidences that the coexistence of multiple homologous genes or proteins is of great significance in the evolution of bacteria [43–45]. Specifically, homologous recombination, mainly mediated by prophage, drives the evolution in some serotype-associated genes, suggesting a critical role of these evolutionary mechanisms in serotype diversification of *Salmonella* [46, 47]. Coincidentally in this current study, these two homologous genes are surrounded by some bacteriophage-related genes (*gtrA* and *gtrB*), which involved in serotype conversion via O-antigen glucosylation [10–14]. The deletion of *SC0368* resulted in the loss of flagella and the double deletion of two homologous genes (*SC0368* and *SC0595*) was involved in LPS core biosynthesis. We boldly speculate that these two serogroup-C1-specific genes may have potential relevance to serotype conversion because flagella and LPS are important factors of serotyping for *Salmonella.*

Otherwise, the differential regulation of orthologous genes or allelic differences in orthologous sequences may have ecological consequences, determining the range of niches a microorganism can occupy [48, 49], such as colonization of the intestine that related with host adaptability. In this current study, *Salmonella* ser. Choleraesuis was chosen as a representative serotype for C1-group mainly because only Choleraesuis contains both these two genes (*SC0368* and *SC0595*). As a primitive transitional serotype, *Salmonella* ser*.* Choleraesuis have a narrow host range and occasionally infect humans and included a high percentage of pseudogenes [50]. The replacement and deletion of these redundant genes potentially allowing more effective interactions with and invasion of host cells to occur or enhancing virulence and motility, which provide a simple evolutionary pathway [50]. Recently, gene loss via deletion, insertional inactivation or truncation has been considered important in the evolution of highly pathogenic *Salmonella* [51, 52]*.* This may be the reason that the △0595 mutant, alike host-restricted *Salmonella* ser. Paratyphi C, only possessing gene *SC0368*, displayed highest cell invasion capacity (Fig. 4). Regrettably, more than half (52.6%) of these differentially expressed genes in the △0595 mutant encoded hypothetical proteins with unknown functions; moreover, the growth and motility of the △0595 mutant were similar to those of the wild-type strain, which make it difficult to find the exactly role of gene *SC0595* played in increasing the cell invasion capacity. Previous studies have shown that flagella-based motility is an important component in the initiation of contact with epithelial cells for *Salmonella* [53], implying that the lower invasion capacity caused by deletion of *SC0368* is due to the lack of flagella. In all, we speculated that these two homologous genes were non-redundant for LPS core biosynthesis and may play different additional roles in motility and virulence.

## Conclusion

Both the transcriptomic and biological data showed that the absence of flagella due to the repressed expression of σ^28^ involved in flagella regulation Class 3 was responsible for the defects in motility of the *SC0368* gene deletion mutants. The increased growth rate observed in the two *SC0368* deletion mutants was correlated with up-regulated expression of genes in cytochrome biogenesis as identified by RNA-Seq data. Moreover, these two C1-specific genes were related to virulence in epithelial cells and potentially involved in serotype diversification. Only the double deletion of the two homologous serogroup C1-specific genes (△0368△0595) resulted in the reduced expression of genes related to side branching in LPS core oligosaccharide biosynthesis. Information from this study can be used to evaluate effects of these specific genes (*SC0368* and *SC0595*) in bacterial structure, physiology and serotype conversion of *Salmonella* serogroup C1*.*

## Methods

### Bacterial strains and growth conditions

Strains and plasmids used in this study are listed in Table S5. A representative strain of *Salmonell*a serogroup C1, *Salmonella* ser. Choleraesuis ATCC 10708, was obtained from the Shanghai Entry-Exit Inspection and Quarantine Bureau of China. The *SC0368* deletion mutant (△0368), the *SC0595* deletion mutant (△0595), the double mutant (△0368△0595) and the corresponding complemented strains (△0368-C, △0595-C and △0368△0595-C) were constructed in this study (Table S5). All of the *Salmonella* and *Escherichia coli* strains were cultured in Luria-Bertani (LB) broth (Oxoid, Cambridge, United Kingdom). When necessary, chloramphenicol and ampicillin were added at concentrations of 35 mg mL^− 1^ and 100 mg mL^− 1^, respectively. The incubation temperature was 37 °C, and all broth cultures were aerated by agitation at 180 rpm.

### Generation of *Salmonella* ser. Choleraesuis ATCC 10708 △0368, △0595, △0368△0595 in-frame deletion mutants and complementation

Deletion mutants △0368, △0595 and △0368△0595 were constructed using a homologous recombination knockout procedure as described previously [54]. Primers were designed according to the principle of overlap extension PCR (Table S6). The fragments of homologous arms were amplified by two rounds of PCR and then were cloned into the pMD_18_-T vector (Takara, Dalian, China) to generate pMD_18_△0368 and pMD_18_△0595. DNA sequencing was carried out to confirm the correct deletion in the plasmids. Both pMD_18_△0368 and pMD_18_△0595 plasmids were digested with *Xba* I and *Sac* I, respectively, and then ligated into suicide vector pRE112. pRE112 vector carries a chloramphenicol resistance gene and a sucrose-sensitivity gene *sacB*. The marker-free in-frame deletion mutants △0368, △0595, and △0368△0595, were screened by double selection as described previously [8]. The resulting plasmids pRE△0368 and pRE△0595 were transformed into *E. coli* SM10λpir by electroporation (2500 V, 5 ms). *E. coli* SM10λpir cells (1 mL ~ 10^6^ CFU mL^− 1^) containing plasmid pRE△0368 or pRE△0595 and 3 mL of *Salmonella* wild-type cells or △0368 cells (~ 10^6^ CFU mL^− 1^) were mixed in a culture flask (quantities to 10 mL) for 8 h at 37 °C to accomplish the conjugation process. Recipient cells were plated onto LA (LB with 1.5% agar) supplemented with chloramphenicol (35 mg mL^− 1^) to select the trans-conjugant strains that contained the plasmid integrated into the *Salmonella* ser. Choleraesuis or *Salmonella* ser. Choleraesuis △0368 genome as a single crossover. After overnight growth of these single crossover strains induced by LB containing 8% (w/v) sucrose, colonies that lost the pRE112 vector were selected.

For the construction of complemented mutant strains, the constructed plasmid pRE△0368-C was transferred into the △0368 mutant, and pRE△0595-C was transferred into △0595 mutant, and pRE△0368-C and pRE△0595-C were successively transferred into the △0368△0595 mutant by electroporation. The resulting strains, *Salmonella* ser. Choleraesuis △0368, △0595, △0368△0595 and complemented strains were confirmed by PCR using primers listed in Table S6.

### RNA isolation, RNA-sequencing, and analysis of differentially expressed genes

For all experiments, a single colony from the overnight cultures was inoculated into LB broth and was grown at 37 °C for 16 h with agitation at 180 rpm. The cultures were diluted 1:100 in fresh LB broth and incubated at 37 °C for 8 h (late exponential phase) with agitation at 180 rpm. Approximately 10^9^ CFU mL^−1^of each strain was pelleted at 5000 g at 4 °C for 10 min. The cell pellets were used for total RNA extraction using a RNeasy Micro Kit (Qiagen, Hilden, Germany) according to the protocol supplied by the manufacturer. Total RNA was extracted from three replicates of each strain. RNA integrity was assessed using a 2100 Bio-analyzer (Agilent, Foster City, CA). Total RNA was further purified by the RNase-Free DNase Set (Qiagen) after quantification. Total RNA samples were stored at − 80 °C until used.

Total RNA samples were submitted to the Shanghai Biotechnology Corporation for mRNA enrichment and subsequent RNA-Seq experiments. Removal of 16S and 23S rRNAs from total RNA was performed using a Ribo-Zero Magnetic Kit (Gram-Negative Bacteria) (Epicentre, Illumina, Madison, WI). The mRNA was used to prepare individually bar-coded (indexed) RNA-Seq libraries with a TruSeq RNA Sample Prep Kit (Illumina, San Diego, CA). Library QC and quantitation were performed on all individual libraries using the Qubit assay (Thermo Fisher Scientific, Waltham, MA) and the Agilent 2100 Bioanalyzer. RNA-Seq libraries were sequenced on a HiSeq2500 platform at the Shanghai Biotechnology Corporation using Version 3 chemistry. Reads were base called and quality filtered with the CASAVA (Consensus Assessment of Sequence and Variance) version 1.8 pipeline (Illumina) to generate single-ended 50-bp reads. The reference genome sequence and functional annotation information of *Salmonella* ser. Choleraesuis were obtained from the NCBI database (NC_006905.1). Mapping was based on a minimal length of 50 bp with an allowance of up to two mismatches, and > 90% of the each read length had to map to the reference sequence for it to be considered a mapped read. After reads were mapped with the CLC Workbench, the total numbers of mapped reads for each gene were counted. These read counts were used for further identification of differentially expressed genes.

The gene expression levels were measured in fragments per kilobase of transcript per million mapped reads (FPKM). The differentially expressed genes (DEGs) were identified using the DEGseq package [55] with the false discovery rate < 0.05 and at least two-fold changes. For the pathway enrichment analysis, all of the identified DEGs were mapped to terms in the KEGG database (*p* < 0.05) (http://www.kegg.jp/kegg/pathway.html#mapping).

### RT-qPCR assays

To measure gene expression levels in different strains, RT-qPCR was carried out using gene-specific primers (Table S2). Total RNA (1.0 μg) was reverse transcribed to generate cDNA as the template for RT-qPCR following the manufacturer’s instructions (Takara, Dalian, China). The RT-qPCR conditions were as follows: 10 μL SYBR mix (Takara, Dalian, China), 1 μL each primer (10 μM), 1 μL cDNA, and 7 μL ddH_2_O. All data in the samples were normalized to the level of the 16S rRNA or *recA* (DNA recombination and repair protein, recombinase A) genes. Three independent technical replicates were carried out for each reaction.

### Growth and motility assays

The wild-type strain and mutants were grown overnight at 37 °C in 5 mL LB broth. Then, 0.1 mL of the cell suspensions (~ 10^8^ CFU mL^− 1^) were transferred into 30 mL flasks containing 10 mL of LB broth and grown at 37 °C with agitation at 180 rpm. Cultures were measured at an optical density of 600 nm (OD_600_) every 2–3 h over the course of 48 h using a spectrophotometer (Tecan, Mannedorf, Switzerland). Three replicate samples were included for each time point. Each experiment was repeated six times.

Bacterial swimming and swarming motility were assayed on LB agar plates containing different concentrations of agar as described previously [56]. Using sterile toothpicks, single colonies from streak plates were stabbed onto the swimming plates (containing 0.3% agar, used to test for motility) and incubated for 24 h. At least six independent colonies were examined for each strain. For swarming motility assays, 5 μL of an overnight culture grown in LB were spotted directly on each plate (containing 0.6% agar, used to test for motility) and allowed to dry for 10 min without the lids. Plates were covered and incubated at 37 °C for 24 h before observation.

### SDS-PAGE analysis of bacterial total protein

Bacterial total proteins were isolated and analyzed by SDS-PAGE with minor modifications [57]. All of the mutant and wild-type strains were grown overnight in LB broth at 37 °C to reach the densities of 10^9^ CFU mL^− 1^. To characterize the total cellular proteins, the bacterial culture (1 mL) was pelleted by centrifugation (12,000×g, 5 min, 4 °C), suspended in SDS-PAGE loading buffer (20% SDS, 25% glycerol, 0.5% β-mercaptoethanol, 0.06 M Tris–HCl, pH 6.8, 0.15% bromophenol blue) and heated at 100 °C for 10 min. All samples were then separated on Ready Gel Precast Tris-HCl polyacrylamide gels with 15 and 5% acrylamide in the separating and stacking gels, respectively (Bio-Rad, Hercules, CA), and then fixed overnight in buffer with 10% acetic acid and 40% methanol. Gels were stained with 2.5 g L^− 1^Coomassie brilliant blue for 2 h. All of the solutions were prepared fresh before use. The experiment was repeated three times.

### Transmission electron microscopy

The flagella and morphological features of the deletion mutants and the wild-type strain were examined by transmission electron microscopy (TEM) as descry [58]. A suspension of each *Salmonella* strain was placed on copper grids, allowed to form a film on the grid for 2 min before the excess solution was removed using absorbent paper, and the grids were dried at room temperature. All samples were examined using a Tecnai G2 spirit Biotwin microscope (FEI, Japan) operated at an accelerating voltage of 120 kV.

### Cell invasion and bacterial cell enumeration using flow cytometry

The human intestinal epithelial cell line Caco-2 (ATCC HTB-37) was cultured in Dulbecco’s Modified Eagle media (DMEM) containing 4 mM glutamine, glucose, 10% (v/v) fetal bovine serum (HyClone, Beijing, China) containing 100 U mL^− 1^penicillin and 100 μg mL^−1^ streptomycin (Life Technologies, Beijing, China) and incubated at 37 °C in the presence of a 5% CO_2_ in a humidified atmosphere. Cells were used between passages 8 and 10. Bacterial invasion was characterized using the gentamicin protection assay as described previously [59]. Data were represented as mean percent recovery of individual *Salmonella* strains in relation to the original inoculum. Experiments were performed in duplicate and repeated three times.

A total of 100 μL each cell lysate that contained bacteria that invaded was re-suspended in 900 μL saline to make a 1-mL bacterial suspension in a 1.5-mL-microcentrifuge tube. For staining, 1 μl of SYTO 9 (ThermoFisher Scientific) that labels live bacterial cells was aliquoted into the microcentrifuge tube. Each sample was incubated in the dark for 15 min at room temperature to allow dye-bacteria binding. Meanwhile, bacterial suspension from the same volume cell lysate without staining by SYTO 9 was as negative control. All samples were evaluated using a LSR II Flow Cytometer (BD biosciences, San Jose, CA, USA). SYTO 9 fluorescence was collected using a 505 nm longpass filter and bandpass filter with transmission at 530/30 nm. The cut-off of SYTO 9 fluorescence value for dye-bacteria binding was chosen based on negative control group at the point that 100% unstained bacteria were covered, and the percentage of bacterial cells above this cut-off was used to characterize the cell invasion ability of each strain.

## Supplementary Information

**Additional file 1: Table S1**. Summary of RNA-Seq data.

**Additional file 2: Table S2**. Primers used in RT-qPCR assays.

**Additional file 3: Table S3**. Down-regulated differentially expressed genes in strain △0368△0595.

**Additional file 4: Table S4**. Up-regulated differentially expressed genes in strain △0368△0595.

**Additional file 5: Table S5**. Strains and plasmids used in this study.

**Additional file 6: Table S6**. Primers used for mutant construction and complementation.

**Additional file 7: Figure S1**. PCR verification of gene deletion mutants. M1:1 Kb ladder; M2: 200 bp ladder; Lanes 1, 5, 9, 13, PCR products using primers SC0368-for and SC0368-rev; Lanes 2, 6, 10, 14 PCR products using primers SC0595-for and SC0595-rev; Lanes 3, 7, 11, 15 PCR products using primers qSC0368-for and qSC0368-rev; Lanes 4, 8, 12, 16 PCR products using primers qSC0595-for and qSC0595-rev. Primer sequences are listed in Table S6.

## Data Availability

All data generated or analyzed during this study are included in this published article and its supplementary information files. All of the raw RNA-Seq data have been deposited in the Gene Expression Omnibus (GEO) with accession number GSE114577.

## Abbreviations

TEM: Transmission electron microcopy
LPS: Lipopolysaccharide
TMS: Transmembrane segments
SDS-PAGE: Sodium dodecylsulphate polyacrylamide gel electrophoresis
LB: Luria-Bertani
FPKM: Fragments per kilobase of transcript per million mapped reads
DEGs: Differentially expressed genes
DMEM: Dulbecco’s Modified Eagle media
GO: Gene Ontology
