# Supplementary material for: Two homologous Salmonella serogroup C1-specific genes are required for flagellar motility and cell invasion
Source: BMC Genomics. 2021 Jul 5;22:507. doi: 10.1186/s12864-021-07759-z (PMC8259012; doi:10.1186/s12864-021-07759-z)
Supplement: Supplementary file 1 — Additional file 1: Table S1. Summary of RNA-Seq data. [file 12864_2021_7759_MOESM1_ESM.docx]

**Table S1 Summary of RNA-Seq data**

| Samples | RNA-seq | | | |  |
| --- | --- | --- | --- | --- | --- |
|  | Q20 Value (%) ^#^ | All reads | Mapped reads | Map rate (%)^*^ | No. of mapped genes |
| Wild-type-1 | 97.94% | 34,106,649 | 33,875,575 | 99.32% | 4601 |
| Wild-type-2 | 97.60% | 33,557,530 | 33,417,512 | 99.58% | 4579 |
| Wild-type-3 | 97.91% | 30,457,294 | 30,303,148 | 99.49% | 4605 |
| △0368-1 | 97.60% | 35,032,456 | 34,642,724 | 98.89% | 4597 |
| △0368-2 | 97.60% | 36,150,614 | 35,814,311 | 99.07% | 4598 |
| △0368-3 | 97.66% | 31,343,793 | 31,129,598 | 99.32% | 4571 |
| △0595-1 | 97.61% | 32,631,108 | 32,319,107 | 99.04% | 4596 |
| △0595-2 | 96.93% | 33,053,595 | 32,910,562 | 99.57% | 4602 |
| △0595-3 | 97.60% | 32,639,977 | 32,455,679 | 99.44% | 4602 |
| △0368△0595-1 | 96.92% | 26,586,404 | 26,275,836 | 98.83% | 4569 |
| △0368△0595-2 | 98.12% | 31,135,906 | 30,780,008 | 98.86% | 4586 |
| △0368△0595-3 | 96.66% | 28,905,791 | 28,604,314 | 98.96% | 4574 |

^#^ Q20=bases of Q>=20 / all bases of sequencing

^*^ Mapping ratio=Mapped reads/All reads
