# Supplementary material for: Two homologous Salmonella serogroup C1-specific genes are required for flagellar motility and cell invasion
Source: BMC Genomics. 2021 Jul 5;22:507. doi: 10.1186/s12864-021-07759-z (PMC8259012; doi:10.1186/s12864-021-07759-z)
Supplement: Supplementary file 2 — Additional file 2: Table S2. Primers used in RT-qPCR assays. [file 12864_2021_7759_MOESM2_ESM.docx]

**Table S2 Primers used in RT-qPCR assays**

| **Gene name** | **Primer Sequences (5′–3′)** | **R^2^/E** | **RNAseq** | **RT-qPCR** | **Use** |
| --- | --- | --- | --- | --- | --- |
| *rfaB* | F:ATAAACGACGCTAATATCTTGC | 0.993/97% | -3.23 | -3.55 | These genes are related to LPS synthesis and were down-regulated in △0368△0595 mutant strain |
|  | R:TTTGGAGGGATGGAAACC |  |  |  |  |
| *rfaI* | F:CGCTTAGTCCACCATTCC | 0.997/97% | -3.54 | -6.49 |  |
|  | R:CCGATTTCTTTGGCTCTG |  |  |  |  |
| *rfaQ* | F:CAGGCTGTCCAAATCTATG | 0.989/102% | -2.84 | -3.29 |  |
|  | R:TACTGGTCTGGCGGGTAA |  |  |  |  |
| *rfaY* | F:CTTAATACGACGCAAGAA | 0.993/99% | -2.27 | -2.35 |  |
|  | R:AAGGCAATGAGATCAGAAT |  |  |  |  |
| *rfaK* | F:TCAGGCAGTTCTGGTTCA | 0.990/98% | -3.38 | -4.37 |  |
|  | R:CTATTCCCAGCGTGTCCT |  |  |  |  |
| *rfaZ* | F:GGCATAGGGCTGGTAGATT | 0.996/97% | -2.71 | -2.61 |  |
|  | R:CTGACGGATGTTCGCTTC |  |  |  |  |
| *nudG* | F:GGCTAATGCCTGATGTTCCA | 0.999/101% | -8.50 | -2.73 | Down-regulated in △0368 mutant strain |
|  | R:GCGAAGAGTTAGGGATTGACG |  |  |  |  |
| *hisD* | F:ATTTCCGCCTCTGACAGTATTA | 0.988/99% | 3.48 | 4.80 | Up-regulated in △0595 mutant strain |
|  | R:GGCTGGGTTTCCACATCTA |  |  |  |  |
| *ibpA* | F:TATCCTGGGCAGTAATCTCC | 0.987/96% | 3.41 | 2.49 |  |
|  | R:GCTACCCTCCGTACAACG |  |  |  |  |
| *pagC* | F:ACCGTTATGAGGATGACTC | 0.990/99% | 4.03 | 8.69 | Up-regulated in △0368△0595 mutant strain |
|  | R:TTAAATGTCGCCTTTACC |  |  |  |  |
| *fliC* | F:TGCTGGCATTGTAGGTTT | 0.999/97% | -316.82 | -883.91 | These genes are related to flagella-mediated motility and were down-regulated in both △0368 and △0368△0595 mutant strains  Up: Relative Expression in △0368  Down: Relative Expression in △0368△0595 |
|  | R:AAGATGGCAAGTATTACGC |  | -250.99 | -624.53 |  |
| *fliA* | F:AACAGGGATGCCAAGACG | 0.994/96% | -113.67 | -53.45 |  |
|  | R:GAACTGGACGATCTGCTACAA |  | -16.78 | -32.91 |  |
| *cheV* | F:GCTGTTATTCCGTCTTGGT | 0.992/101% | -74.81 | -44.94 |  |
|  | R:GGTCTTCAGATTGGTGGC |  | -11.67 | -16.92 |  |
| *cheY* | F:TCACATGCCCAGTTTCTCA | 0.989/98% | -98.94 | -93.09 |  |
|  | R:ACCATGCGTCGTATCGTG |  | -6.76 | -5.10 |  |
| *motA* | F:CGCATAGCCGTTCAGATT | 0.991/97% | -21.01 | -10.39 |  |
|  | R:TGGATTCATTTCACCGTTAG |  | -17.56 | -15.25 |  |
| *flgM* | F:GCGAATGAGCGAGTCTGC | 0.991/96% | -88.74 | -97.43 |  |
|  | R:TGACCGTACCTCACCTTTGA |  | -5.44 | -13.29 |  |
| *yhjH* | F:CACTCTTCCAGCGTCTCC | 0.993/103% | -22.13 | -19.84 |  |
|  | R:TGAGCGAAGTGCGTTATG |  | -3.06 | -8.78 |  |
| *sdiA* | F:TGCTACGGGAGACAGATAA | 0.995/97% | -13.81 | -2.83 |  |
|  | R:ATTTCAGGCAGGGTCATT |  | -10.90 | -3.44 |  |
| *recA* | F:CGGGTTACCGAACATCAC | 0.992/95% |  |  | Reference genes |
|  | R:TTTCACTGGACATCGCACT |  |  |  |  |
| *16S* | F:CCCCTGGACAAAGACTGA | 0.997/98% |  |  |  |
|  | R:CTAATCGCTGGCAACAAA |  |  |  |  |
