# Supplementary material for: Two homologous Salmonella serogroup C1-specific genes are required for flagellar motility and cell invasion
Source: BMC Genomics. 2021 Jul 5;22:507. doi: 10.1186/s12864-021-07759-z (PMC8259012; doi:10.1186/s12864-021-07759-z)
Supplement: Supplementary file 3 — Additional file 3: Table S3. Down-regulated differentially expressed genes in strain △0368△0595. [file 12864_2021_7759_MOESM3_ESM.docx]

**Table S3 Down-regulated differentially expressed genes in strain △0368△0595**

| Gene name | Description | Relative gene expression [Fold change] | | | | |
| --- | --- | --- | --- | --- | --- | --- |
|  |  | **△0368△0595** | | **△0368** | **△0595** | |
| **fliC* | flagellin | 0.003156 | 0.003984 | | |  |
| **SC0368* | hypothetical protein | 0.003959 | 0.008457 | | |  |
| **SCPS48* | pseudo | 0.004502 | 0.019855 | | |  |
| **cheM* | methyl accepting chemotaxis protein II, aspartate sensor-receptor | 0.00727 | 0.088214 | | |  |
| **cheR* | chemotaxis methyltransferase CheR | 0.007692 | 0.073845 | | |  |
| **cheV* | chemotaxis signal transduction protein | 0.008797 | 0.059577 | | |  |
| **motB* | flagellar motor protein MotB | 0.009488 | 0.18416 | | |  |
| **yhjH* | diguanylate phosphodiesterase | 0.011273 | 0.183777 | | |  |
| **motA* | flagellar motor protein MotA | 0.011372 | 0.166312 | | |  |
| **cheY* | chemotaxis regulatory protein CheY | 0.013367 | 0.085679 | | |  |
| **cheW* | purine-binding chemotaxis protein | 0.013543 | 0.128391 | | |  |
| **cheB* | chemotaxis-specific methylesterase | 0.014051 | 0.138133 | | |  |
| **cheA* | chemotaxis protein CheA | 0.014211 | 0.122824 | | |  |
| **cheZ* | chemotaxis regulator CheZ | 0.018975 | 0.093346 | | |  |
| *SC1963* | hypothetical protein | 0.019263 |  | | |  |
| *^#^SC0595* | hypothetical protein | 0.022391 |  | | | 0.042674 |
| **flgN* | for FlgK and FlgL | 0.029784 | 0.056887 | | |  |
| **tcp* | methyl-accepting transmembrane citrate/phenol chemoreceptor | 0.032267 | 0.297117 | | |  |
| **mcp2* | methyl-accepting chemotaxis protein | 0.032569 | 0.261263 | | |  |
| *SC2703* | hypothetical protein | 0.034453 | | | | |
| *SC4386* | hypothetical protein | 0.035565 | | | | |
| **tsr* | methyl-accepting chemotaxis protein I, serine sensor receptor | 0.036943 |  | | |  |
| **trg* | methyl-accepting chemotaxis protein III, ribose and galactose sensor receptor | 0.037447 |  | | |  |
| *ycgR* | hypothetical protein | 0.04472 | | | | |
| **flgM* | anti-sigma-28 factor FlgM | 0.045945 | 0.05495 | | |  |
| **sdiA* | DNA-binding transcriptional activator SdiA | 0.051117 | 0.368825 | | |  |
| **flgK* | flagellar hook-associated protein FlgK | 0.07001 | 0.096679 | | |  |
| **ynjH* | hypothetical protein | 0.070088 | 0.096679 | | |  |
| **^#^fliA* | flagellar biosynthesis sigma factor | 0.08159 | 0.103725 | | | 0.361662 |
| **fliD* | flagellar capping protein | 0.086567 | 0.128325 | | |  |
| **nudG* | pyrimidine (deoxy)nucleoside triphosphate pyrophosphohydrolase | 0.088136 | 0.128325 | | |  |
| *yjdB* | cell division protein | 0.088992 | | | | |
| *yfbK* | von Willebrand factor A | 0.097521 | | | | |
| *fliZ* | flagella biosynthesis protein FliZ | 0.098563 |  | | |  |
| **flgL* | flagellar hook-associated protein FlgL | 0.108689 | 0.15773 | | |  |
| **fliS* | flagellar protein FliS | 0.137607 | 0.200468 | | |  |
| *fliT* | flagellar biosynthesis protein FliT | 0.138014 |  | | |  |
| **mcp* | methyl-accepting chemotaxis protein | 0.147189 | 0.200468 | | |  |
| *SC3096* | ATP-dependent RNA helicase-like protein | 0.168907 | | | | |
| *cysP* | thiosulfate transporter subunit | 0.189256 | | | | |
| *yhhP* | sulfur transfer protein SirA | 0.190102 | | | | |
| *modA* | molybdate transporter periplasmic protein | 0.203026 | | | | |
| *^a^rfaY* | lipopolysaccharide core biosynthesis protein | 0.209202 | | | | |
| *^a^rfaQ* | lipopolysaccharide core biosynthesis protein | 0.210331 | | | | |
| *rpmE* | 50S ribosomal protein L31 | 0.210721 | | | | |
| *SCTRNA67* | tRNA | 0.215478 | | | | |
| *modC* | molybdate transporter ATP-binding protein | 0.225428 | | | | |
| *^a^rfaI* | UDP-D-galactose:(glucosyl)lipopolysaccharide-alpha-1,3-D-galactosyltransferase | 0.226319 | | | | |
| *^a^rfaB* | UDP-D-galactose:(glucosyl)lipopolysaccharide-1,6-D-galactosyltransferase | 0.228627 | | | | |
| *slsA* | inner membrane protein | 0.229063 | | | | |
| *prpA* | hypothetical protein | 0.231056 | | | | |
| *SC2468* | hypothetical protein | 0.232238 | | | | |
| *yhdV* | outer membrane lipoprotein | 0.233958 | | | | |
| *yaiB* | hypothetical protein | 0.249357 | | | | |
| *mac* | hypothetical protein | 0.250399 | | | | |
| *SC2210* | hypothetical protein | 0.252625 | | | | |
| *yeeI* | hypothetical protein | 0.25388 | | | | |
| *SC3538* | hypothetical protein | 0.258671 | | | | |
| *pdxH* | pyridoxamine 5'-phosphate oxidase | 0.268385 | | | | |
| *modB* | molybdate ABC transporter permease | 0.272547 | | | | |
| *groES* | co-chaperonin GroES | 0.273386 | | | | |
| *cysK* | cysteine synthase A | 0.293303 | | | | |
| *smpB* | SsrA-binding protein | 0.29806 | | | | |
| *ycfF* | purine nucleoside phosphoramidase | 0.299583 | | | | |
| *ihfB* | integration host factor subunit beta | 0.299656 | | | | |
| *ygbE* | hypothetical protein | 0.299863 | | | | |
| *slp* | outer membrane protein | 0.315414 | | | | |
| *yoaG* | hypothetical protein | 0.320085 | | | | |
| *yhdN* | hypothetical protein | 0.320163 | | | | |
| *SC2092* | hypothetical protein | 0.325619 | | | | |
| *seqA* | replication initiation regulator SeqA | 0.326157 | | | | |
| *SC2988* | hypothetical protein | 0.329668 | | | | |
| *ydhF* | aldo/keto reductase | 0.32998 | | | | |
| *SC1812* | hypothetical protein | 0.331805 | | | | |
| *ypeC* | hypothetical protein | 0.334678 | | | | |
| *gltL* | glutamate/aspartate ABC transporter ATP-binding protein | 0.337693 | | | | |
| *fur* | ferric uptake regulator | 0.340579 | | | | |
| *folE* | GTP cyclohydrolase I | 0.345099 | | | | |
| *glnK* | nitrogen regulatory protein P-II 2 | 0.351664 | | | | |
| *celC* | PTS system N,N'-diacetylchitobiose-specific transporter subunit IIA | 0.354938 | | | | |
| *zntR* | zinc-responsive transcriptional regulator | 0.360155 | | | | |
| *^#^gltJ* | glutamate/aspartate ABC transporter | 0.361158 |  | | | 0.425947 |
| *SC1698* | hypothetical protein | 0.373704 | | | | |
| *yeaA* | methionine sulfoxide reductase B | 0.376253 | | | | |
| *ddl* | D-alanyl-alanine synthetase A | 0.376716 | | | | |
| *pgsA* | phosphatidylglycerophosphate synthetase | 0.376874 | | | | |
| *cfa* | cyclopropane-fatty-acyl-phospholipid synthase | 0.37846 | | | | |
| *yhgI* | DNA uptake protein | 0.378651 | | | | |
| *gltK* | glutamate/aspartate ABC transporter | 0.381103 | | | | |
| *dadA* | D-amino acid dehydrogenase small subunit | 0.382262 | | | | |
| *^#^hns* | global DNA-binding transcriptional dual regulator H-NS | 0.382641 |  | | | 0.435074 |
| *yqjI* | transcriptional regulator | 0.389136 | | | | |
| *^#^yobF* | hypothetical protein | 0.398913 |  | | | 0.34537 |
| *yodD* | hypothetical protein | 0.400376 | | | | |
| *sdhC* | succinate dehydrogenase cytochrome b556 large membrane subunit | 0.410078 | | | | |
| *ybjP* | lipoprotein | 0.415582 | | | | |
| *^#^ybhQ* | hypothetical protein | 0.419787 |  | | | 0.372804 |
| *SC2882* | hypothetical protein | 0.427459 | | | | |
| *yfiA* | translation inhibitor protein RaiA | 0.430368 | | | | |
| *sfsA* | sugar fermentation stimulation protein A | 0.432528 | | | | |
| *ybhR* | ABC transporter membrane protein | 0.433145 | | | | |
| *^a^rfaK* | hexose transferase, lipopolysaccharide core biosynthesis | 0.43498 | | | | |
| *^a^rfaZ* | lipopolysaccharide core biosynthesis protein | 0.437147 | | | | |
| *SC3583* | hypothetical protein | 0.437659 | | | | |
| *trxC* | thioredoxin | 0.438267 | | | | |
| *SC0937* | hypothetical protein | 0.4385 | | | | |
| *lipA* | lipoyl synthase | 0.440208 | | | | |
| *fliB* | flagellin methylation protein | 0.440356 | | | | |
| *SC1895* | hypothetical protein | 0.441778 | | | | |
| *yfcF* | glutathione-S-transferase | 0.443725 | | | | |
| *vacJ* | lipoprotein | 0.445546 | | | | |
| *rpoD* | RNA polymerase sigma factor RpoD | 0.445874 |  | | |  |
| *^#^cspC* | cold shock-like protein CspC | 0.446117 |  | | | 0.339589 |
| *manC* | phosphomannomutase | 0.446764 | | | | |
| *sanA* | hypothetical protein | 0.448812 | | | | |
| *^*^osmB* | lipoprotein | 0.451396 | 0.497226 | | |  |
| *SC2404* | hypothetical protein | 0.451983 | | | | |
| *cbpA* | curved DNA-binding protein CbpA | 0.453582 | | | | |
| *SC1146* | hypothetical protein | 0.454743 | | | | |
| *SC2097* | hypothetical protein | 0.457154 | | | | |
| *SC2855* | hypothetical protein | 0.45788 | | | | |
| *glpE* | thiosulfate sulfurtransferase | 0.457918 | | | | |
| *hisJ* | histidine ABC transporter ATP-binding protein | 0.459298 | | | | |
| *yoaH* | hypothetical protein | 0.459787 | | | | |
| *SC3139* | hypothetical protein | 0.460125 | | | | |
| *mrdB* | cell wall shape-determining protein | 0.465303 | | | | |
| *speB* | agmatinase | 0.471013 | | | | |
| *dadX* | alanine racemase | 0.473932 | | | | |
| *SC3730* | hypothetical protein | 0.474505 | | | | |
| *yccD* | chaperone-modulator protein CbpM | 0.47611 | | | | |
| *SC1383* | hypothetical protein | 0.478347 | | | | |
| *SC3103* | hypothetical protein | 0.482509 | | | | |
| *sdaA* | L-serine deaminase I/L-threonine deaminase I | 0.488401 | | | | |
| *dnaG* | DNA primase | 0.489351 | | | | |
| *SC4375* | hypothetical protein | 0.49191 | | | | |
| *dksA* | RNA polymerase-binding transcription factor | 0.492528 | | | | |
| *rnc* | ribonuclease III | 0.494118 | | | | |
| *arcA* | two-component response regulator | 0.494709 | | | | |
| *SCTRNA10* | tRNA | 0.498719 | | | | |

**^*^ Down-regulated differential expression genes shared in △0368 and △0368△0595**

**^#^ Down-regulated differential expression genes shared in △0595 and △0368△0595**

**^a^ Down-regulated differential expression genes in lipopolysaccharide core biosynthesis**
