# Supplementary material for: Two homologous Salmonella serogroup C1-specific genes are required for flagellar motility and cell invasion
Source: BMC Genomics. 2021 Jul 5;22:507. doi: 10.1186/s12864-021-07759-z (PMC8259012; doi:10.1186/s12864-021-07759-z)
Supplement: Supplementary file 4 — Additional file 4: Table S4. Up-regulated differentially expressed genes in strain △0368△0595. [file 12864_2021_7759_MOESM4_ESM.docx]

**Table S4 Up-regulated differentially expressed genes in strain △0368△0595**

| Gene name | Description | Relative gene expression[fold change] | | | | | |  |  |
| --- | --- | --- | --- | --- | --- | --- | --- | --- | --- |
|  |  | **△0368△0595** | | **△0368** | **△0595** | | |  |  |
| *^#^SC2696* | hypothetical protein | 7.15 |  | | | | 5.79 |  |  |
| **pagC* | PhoP regulated protein: reduced macrophage survival | 4.03 | 2.56 | | | |  |  |  |
| *^#^*SC3193* | hypothetical protein | 4.02 | 2.05 | | | | 3.65 |  |  |
| *ugtL* | hypothetical protein | 3.95 | | | | | |  |  |
| *SC1597* | inner membrane protein | 3.94 | | | | | |  |  |
| **rpsV* | 30S ribosomal subunit S22 | 3.62 | 2.86 | | |  | |  |  |
| **pagD* | PhoP regulated protein | 3.54 | 2.75 | | |  | |  |  |
| **dcdA* | diaminopimelate decarboxylase | 3.43 | 2.17 | | |  | |  |  |
| *ssaR* | type III secretion system protein | 3.42 | | | | | |  |  |
| *mgtB* | Mg2+ transport protein | 3.28 | | | | | |  |  |
| **garR* | tartronate semialdehyde reductase | 3.23 | 2.23 | | |  | |  |  |
| **yciG* | hypothetical protein | 3.22 | 3.14 | | |  | |  |  |
| *^#^SC3553* | hypothetical protein | 3.21 |  | | | 4.38 | |  |  |
| *yjbE* | hypothetical protein | 3.21 | | | | | |  |  |
| *rmf* | ribosome modulation factor | 3.07 | | | | | |  |  |
| **SCPS4* | pseudo | 3.05 | 2.41 | | |  | |  |  |
| **SC4215* | entericidin B | 3.01 | 2.77 | | |  | |  |  |
| *^#^*SCTRNA83* | tRNA | 2.96 | 2.96 | | | 2.76 | |  |  |
| *csiE* | stationary phase inducible protein CsiE | 2.91 | | | | | |  |  |
| *gudT* | MFS superfamily, D-glucarate permease | 2.91 | | | | | |  |  |
| *SC2360* | hypothetical protein | 2.90 | | | | | |  |  |
| *^*#^SCTRNA84* | tRNA | 2.88 | 2.23 | | | 2.42 | |  |  |
| *prpA* | hypothetical protein | 2.87 | | | | | |  |  |
| **ccmD* | heme exporter protein C, cytochrome c-type biogenesis protein | 2.82 | 2.35 | | |  | |  |  |
| **SC1273* | hypothetical protein | 2.76 | 2.10 | | |  | |  |  |
| *garL* | alpha-dehydro-beta-deoxy-D-glucarate aldolase | 2.76 | | | | | |  |  |
| *SC2361* | amino acid transporter | 2.74 | | | | | |  |  |
| *sopD* | secreted protein SopD-like protein | 2.73 | | | | | |  |  |
| *SC1850* | inner membrane protein | 2.72 | | | | | |  |  |
| **ccmC* | heme ABC exporter | 2.72 | 2.59 | | |  | |  |  |
| *^#^SCTRNA85* | tRNA | 2.69 |  | | | 2.47 | |  |  |
| *SCTRNA25* | tRNA | 2.67 | | | | | |  |  |
| **^#^glgS* | glycogen synthesis protein GlgS | 2.63 | 2.68 | | | 2.21 | |  |  |
| *yehW* | ABC-type proline/glycine betaine transport systems, permease component | 2.60 | | | | | |  |  |
| *SC1257* | lysozyme inhibitor | 2.60 | | | | | |  |  |
| **ccmA* | cytochrome c biogenesis protein CcmA | 2.54 | 3.08 | | |  | |  |  |
| *SC3718* | toxic peptide TisB | 2.53 | | | | | |  |  |
| **SC0403* | inner membrane protein | 2.53 | 2.79 | | |  | |  |  |
| *SC3612* | hypothetical protein | 2.52 | | | | | |  |  |
| *nanT* | sialic acid transporter | 2.52 | | | | | |  |  |
| *rbsB* | D-ribose transporter subunit RbsB | 2.52 | | | | | |  |  |
| **ctjC* | hypothetical protein | 2.50 | 2.39 | | |  | |  |  |
| *mgtC* | Mg2+ transport protein | 2.46 | | | | | |  |  |
| **fruA* | PTS system fructose-specific transporter subunit IIBC | 2.43 | 2.44 | | |  | |  |  |
| *leuD* | hypothetical protein | 2.43 | | | | | |  |  |
| *ssaN* | type III secretion system ATPase | 2.42 | | | | | |  |  |
| *SCPS116* | pseudo | 2.40 | | | | | |  |  |
| *pgtE* | outer membrane protease | 2.40 | | | | | |  |  |
| *^#^SC3192* | hypothetical protein | 2.39 |  | | | 2.31 | |  |  |
| *cydB* | cytochrome BD2 subunit II | 2.37 | | | | | |  |  |
| *yjfN* | hypothetical protein | 2.37 | | | | | | 2.261412 |  |
| **hypO* | hydrogenase 2 small subunit | 2.37 | 2.20 | | |  | |  |  |
| **nrfA* | cytochrome c552 | 2.35 | 2.37 | | |  | |  |  |
| **ccmB* | heme ABC exporter | 2.35 | 2.14 | | |  | |  |  |
| *pspG* | phage shock protein G | 2.34 | | | | | |  |  |
| *cdaR* | carbohydrate diacid transcriptional activator CdaR | 2.29 | | | | | |  |  |
| **yrbL* | hypothetical protein | 2.28 | 2.40 | | |  | |  |  |
| *dcuA* | anaerobic C4-dicarboxylate transporter | 2.24 | | | | | |  |  |
| *bglA* | glycosyl hydrolase family protein | 2.24 | | | | | |  |  |
| *pipB* | pentapeptide repeat-containing protein | 2.22 | | | | | |  |  |
| *lpp* | links outer and inner membranes | 2.22 | | | | | |  |  |
| *SC1489* | hypothetical protein | 2.21 | | | | | |  |  |
| *nrfD* | nitrate reductase, formate dependent | 2.21 |  | | |  | |  |  |
| *SC3894* | hypothetical protein | 2.18 | | | | | |  |  |
| *SC0905* | hypothetical protein | 2.16 | | | | | |  |  |
| *SCPS60* | pseudo | 2.16 | | | | | |  |  |
| *sseA* | secretion system effector protein SseA | 2.15 | | | | | |  |  |
| **SCPS123* | pseudo | 2.14 | 2.40 | | |  | |  |  |
| *^#^grk* | hypothetical protein | 2.13 |  | | | 2.90 | |  |  |
| *SC2709* | inner membrane protein | 2.13 | | | | | |  |  |
| *trpD* | bifunctional glutamine amidotransferase/anthranilate phosphoribosyltransferase | 2.08 | | | | | |  |  |
| *SCV19* | hypothetical protein | 2.08 | | | | | |  |  |

*** Up-regulated differential expression genes shared in △0368 and △0368△0595**

**# Up-regulated differential expression genes shared in △0595 and △0368△0595**
