# Supplementary material for: Two homologous Salmonella serogroup C1-specific genes are required for flagellar motility and cell invasion
Source: BMC Genomics. 2021 Jul 5;22:507. doi: 10.1186/s12864-021-07759-z (PMC8259012; doi:10.1186/s12864-021-07759-z)
Supplement: Supplementary file 5 — Additional file 5: Table S5. Strains and plasmids used in this study. [file 12864_2021_7759_MOESM5_ESM.docx]

**Table S5 Strains and plasmids used in this study**

| **Strain or plasmid** | **Comment(s)** | **Source/reference** |
| --- | --- | --- |
| *S.* Choleraesuis ATCC10708 | Wild type, *Salmonella* serogroup C1, Standard strain | Shanghai Entry-Exit Inspection and Quarantine Bureau of China |
| △0368  △0595 | *SC0368* deletion mutant of *S.* Choleraesuis ATCC10708 by pRE112-0368  *SC0595* deletion mutant of *S.* Choleraesuis ATCC10708 by pRE112-0595 | This study  This study |
| △0368△0595 | *SC0595* deletion mutant of *S.* Choleraesuis △0368 by pRE112-0595 | This study |
| △0368-C | Complemented strain of △0368 by pRE112-0368-C | This study |
| △0595-C | Complemented strain of △0595 by pRE112-0595-C | This study |
| △0368△0595-C | Complemented strain of △0368△0595 successively by pRE112-0368-C and pRE112-0595-C | This study |
| *E. coli* TG1 | *sup*E, *hsd*Δ5, *thi*Δ(lac-proAB), F′[*tra*D36, *pro*AB+, *lac*Iq, *lac*ZΔM15] | Laboratory stock |
| *E. coli* SM10 (*λpir*) | *thi thr*-1 *leu*6 *pro*A2 *his*-4 *arg* E2 *lac*Y1 *galK*2, *ara*14*xy*l5 *supE*44, *λpir* | Laboratory stock |
| pMD_18_-T | Cloning vector, Amp^r^ | TaKaRa, Japan |
| pRE112 | pGP704 suicide plasmid, *pir* dependent, *ori*T, *ori*V, *sac*B, Cm^r^ | Laboratory stock |
| pREΔ0368 | pRE112 containing a 1439 bp *SC0368*-deletion PCR product (742 bp of sequence upstream and 694 bp of sequence downstream of *SC0368* generated by overlap extension PCR); used to generate strain △0368 | This study |
| pREΔ0595 | pRE112 containing a 1350 bp *SC0595*-deletion PCR product (600 bp of sequence upstream and 750 bp of sequence downstream of *SC0595* generated by overlap extension PCR); used to generate strain △0595 and △0368△0595 | This study |
| pREΔ0368-C | pRE112 containing a 2892 bp *SC0368*-complemented PCR product; used to generate strain △0368-C and △0368△0595-C |  |
| pREΔ0595-C | pRE112 containing a 2804 bp *SC0595*- complemented PCR product; used to generate strain △0595-C and △0368△0595-C |  |
