# Supplementary material for: Two homologous Salmonella serogroup C1-specific genes are required for flagellar motility and cell invasion
Source: BMC Genomics. 2021 Jul 5;22:507. doi: 10.1186/s12864-021-07759-z (PMC8259012; doi:10.1186/s12864-021-07759-z)
Supplement: Supplementary file 6 — Additional file 6: Table S6. Primers used for mutant construction and complementation. [file 12864_2021_7759_MOESM6_ESM.docx]

**Table S6 Primers used for mutant construction and complementation**

| **Primes** | **Primer sequences** | | **Usage** |
| --- | --- | --- | --- |
| SC0368-for | 5’-GC**TCTAGA** GGACGAAGACGCTTATCC-3’ (*Xba* I) | △0368 mutant construction and △0368 and △0368 △0595 complementation | |
| SC0368-rev | 5’-C**GAGCTC** GACGTAGATTTGCAAGACCC-3’ (*Sac* I) |  |  |
| SC0368-int-rev | 5’-**GGGAAGAACA AAAAGGCGC**GAAAGCGCC-3’ | △0368 mutant construction | |
| SC0368-int-for | 5’-**CGCGCCTTTT TGTTCTTCCC**CTTCAAAATA-3’ |  |  |
| SC0595-for | 5’-GC**TCTAGA** CTGGGTATCAACACCGAAGG -3’(*Xba* I) | △0595 and △0368 △0595 mutants construction and △0595 and △0368 △0595 complementation | |
| SC0595-rev | 5’-C**GAGCTC** GATGCGGTCATTCCTATT -3’ (*Sac* I) |  |  |
| SC0595-int-rev | 5’- **GGAAGAATAGTCAACAAGGA**GCCTGTCCAT -3’ | △0595 and △0368 △0595 mutants construction | |
| SC0595-int-for | 5’- **TCCTTGTTGACTATTCTTCC**CCTTCAAAA -3’ |  |  |
| qSC0368-for | 5’- ACGCTCCGTCTATTTCTT -3’ | Reconfirm of mutant constructs by PCR and expression analysis by RT-qPCR | |
| qSC0368-rev | 5’-AACCTCGGTGATGGCTAC-3’ |  |  |
| qSC0595-for | 5’-CTACTCTATAACAATCCTTGCA-3’ |  |  |
| qSC0595-rev | 5’-CACGCTATCTAAATTCACAA-3’ |  |  |
| M13F | 5’-GGTTTTCCCAGTCACGAC-3’ | Sequencing | |
| M13R | 5’-AGCGGATAACAATTTCACAC-3’ |  |  |
