# Supplementary figures and images for: Two homologous Salmonella serogroup C1-specific genes are required for flagellar motility and cell invasion
Source: BMC Genomics. 2021 Jul 5;22:507. doi: 10.1186/s12864-021-07759-z (PMC8259012; doi:10.1186/s12864-021-07759-z)

**Fig. S1**

**
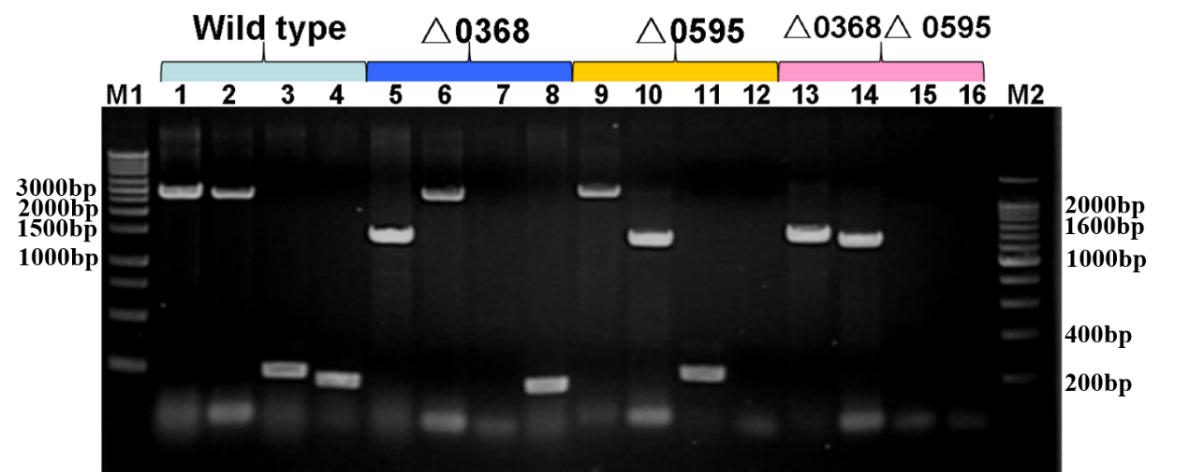
**

Supplement: Supplementary file 7 — Additional file 7: Figure S1. PCR verification of gene deletion mutants. M1:1 Kb ladder; M2: 200 bp ladder; Lanes 1, 5, 9, 13, PCR products using primers SC0368-for and SC0368-rev; Lanes 2, 6, 10, 14 PCR products using primers SC0595-for and SC0595-rev; Lanes 3, 7, 11, 15 PCR products using primers qSC0368-for and qSC0368-rev; Lanes 4, 8, 12, 16 PCR products using primers qSC0595-for and qSC0595-rev. Primer sequences are listed in Table S6. [file 12864_2021_7759_MOESM7_ESM.docx]
